# Negative and positive childhood experiences across developmental periods in psychiatric patients with different diagnoses – an explorative study

**DOI:** 10.1186/1471-244X-4-40

**Published:** 2004-11-26

**Authors:** Evangelia Saleptsi, Dana Bichescu, Brigitte Rockstroh, Frank Neuner, Margarete Schauer, Karl Studer, Klaus Hoffmann, Thomas Elbert

**Affiliations:** 1 Department of Psychology, University of Konstanz, Fach D-25, 78457 Konstanz, Germany; 2 Department of Psychology, University of Jassy, Jassy, Romania; 3 Center for Psychiatry Reichenau, Konstanz, Germany; 4 Psychiatric Hospital Münsterlingen, Münsterlingen, Switzerland

## Abstract

**Background:**

A high frequency of childhood abuse has often been reported in adult psychiatric patients. The present survey explores the relationship between psychiatric diagnoses and positive and negative life events during childhood and adulthood in psychiatric samples.

**Methods:**

A total of 192 patients with diagnoses of alcohol-related disorders (n = 45), schizophrenic disorders (n = 52), affective disorders (n = 54), and personality disorders (n = 41) completed a 42-item self-rating scale (Traumatic Antecedents Questionnaire, TAQ). The TAQ assesses personal positive experiences (competence and safety) and negative experiences (neglect, separation, secrets, emotional, physical and sexual abuse, trauma witnessing, other traumas, and alcohol and drugs abuse) during four developmental periods, beginning from early childhood to adulthood. Patients were recruited from four Psychiatric hospitals in Germany, Switzerland, and Romania; 63 subjects without any history of mental illness served as controls.

**Results:**

The amount of positive experiences did not differ significantly among groups, except for safety scores that were lower in patients with personality disorders as compared to the other groups. On the other side, negative experiences appeared more frequently in patients than in controls. Emotional neglect and abuse were reported in patients more frequently than physical and sexual abuse, with negative experiences encountered more often in late childhood and adolescence than in early childhood. The patients with alcohol-related and personality disorders reported more negative events than the ones with schizophrenic and affective disorders.

**Conclusions:**

The present findings add evidence to the relationship between retrospectively reported childhood experiences and psychiatric diagnoses, and emphasize the fact that a) emotional neglect and abuse are the most prominent negative experiences, b) adolescence is a more 'sensitive' period for negative experiences as compared to early childhood, and c) a high amount of reported emotional and physical abuse occurs in patients with alcohol-related and personality disorders respectively.

## Background

It is difficult to assess the impact of childhood traumatic events on the psychiatric disorders in adulthood, as neither prospective research studies, nor experimental approaches are possible. Nevertheless, an increasing number of retrospective reports suggest that psychiatric disorders may be related to childhood psychological traumas such as neglect, physical or emotional abuse [1-6]. In particular, significant correlations between the severity of psychiatric symptoms and that of stressful and traumatic experiences during childhood were found [7-12]. Reports of physical and sexual abuse in childhood are more frequent in psychiatric patients than in the healthy population [13-16]; among these are patients diagnosed with affective disorders [17-19], somatization disorders [20-22], borderline personality disorders [3,7,23-25], substance-related disorders [26-28], and schizophrenic disorders [29-31]. Specifically, several studies have documented high rates of trauma in individuals with severe mental illness [32]. For a sample of schizophrenic women, Friedmann and Harisson (1984) reported that 60% of them had suffered childhood sexual abuse [33]. Abused patients displayed more pronounced symptoms such as hallucinations [34,35] and delusions [36].

Any conclusion to such reports, however, must be drawn by taking into consideration that the validity of childhood memories, particularly in psychiatric patients, may be questioned, as the range of childhood traumas indexed in these studies is generally limited, and often only childhood sexual abuse is targeted. Moreover, the observed relationships are correlational in nature, and do not justify the conclusion that childhood trauma favors the development of psychiatric disorders. Antecedents of developing psychopathology may also provoke certain parental behavior. Also, a third variable, such as social conditions, may have caused both childhood abuse and later pathological development. Another notable finding is that the prevalence rates of antecedent traumatic events vary considerably across studies. This may be due to different definitions of abuse which include more detailed [13] or more global [23] descriptions. Furthermore, the amount of psychosocial elements such as neglect, family disturbance, the nature of preexisting and subsequent attachment patterns, special competencies, etc., is difficult to be assessed or taken into account. Only a limited number of studies [37,38] have so far included control groups, allowing one to compare self-reports of abusive sexual experiences during childhood in psychiatric patients to those in the healthy population. There is also a lack of research studies that assess these issues within different cultural backgrounds.

The present study sets out to evaluate reported positive and negative life events from early childhood to adulthood in psychiatric patients. We addressed some of the above-mentioned problems by examining abuse histories across a range of several psychiatric diagnoses within a controlled cross-national design. We sought to examine whether (a) negative life experiences are positively associated with psychiatric diagnoses in adulthood, and (b) early childhood and adolescence were 'sensitive periods', that is, whether psychiatric diagnoses were more closely related to negative experience in these developmental periods.

The present study includes a German/Swiss and a Romanian psychiatric group, in order to determine whether reports vary between cultural backgrounds.

## Methods

### Subjects

Patients were recruited from four Psychiatric Hospitals within two different cultural settings, Switzerland/Southern Germany versus the Moldavia region in Romania: the Center for Psychiatry Reichenau and the Center for Psychiatry Weissenau in Germany, the Psychiatric Hospital Münsterlingen in Switzerland, and the Psychiatric Hospital "Socola", Jassy in Romania. A total of 192 psychiatric inpatients (98 German and 94 Romanian psychiatric patients, range 18–78 years) filled in the questionnaire. Sixty-three control subjects without any history of psychiatric diagnosis were recruited from the clinical staff and the university employees (Konstanz in Germany, Jassy in Romania) as controls (38 Germans and 25 Romanians). The control subjects have been simply inquired whether they had any stationary hospitalization in the psychiatry; no further assessments have been done. After a full explanation of the study, written informed consent was obtained from all subjects.

By considering the clinician-made diagnoses which were written down from the medical files available in the psychiatric clinics the patients were recruited from, the patients were distributed in four diagnostic groups: alcohol-related disorders (n = 45), schizophrenic disorders (n = 52), affective disorders (n = 54), and personality disorders (n = 41). At all psychiatric clinics in Germany/Switzerland and Romania the diagnoses were made according to the ICD-10 criteria. Within our patient groups, the following lifetime mental disorders were assessed by using the ICD-10: alcohol-related disorders (dependence syndrome, psychotic and unspecified mental disorders due to the use of alcohol), schizophrenic disorders (paranoid schizophrenia, schizoaffective disorder, and undifferentiated schizophrenia), affective disorders (bipolar depressive disorders, recurrent depressive disorder, cyclothymia, and dysthymia), and personality disorders (borderline, schizoid, paranoid, histrionic, dissocial, and dependent personality disorder respectively). A few patients within our sample were diagnosed with comorbid symptoms: 4 patients with affective disorders had symptoms of substance abuse and 7 of them had anxiety symptoms; also, within the schizophrenic disorders group, 2 patients had symptoms of alcohol abuse and 6 had depressive symptoms. There were also patients who received two diagnoses: one of which was a personality disorder (i.e., 5 patients with affective disorders, 7 with alcohol-related disorders, and 2 with schizophrenic disorders). In these cases, we considered the other diagnosis for the distribution into the diagnostic groups.

Table 1 summarizes the demographical characteristics of all subjects. The patient groups were similar with respect to the psychiatric history. There were differences among groups concerning gender distribution, age, and education. The gender-distribution differences among groups were due to the high number of women within the control and the affective disorders groups. With regard to the noted age differences, the patients with affective and alcohol-related disorders respectively had higher mean age as compared to all the other groups. The education differences among groups are only due to the lower educational level in patients with alcohol-related disorders. Romanian patients with affective disorders had a longer psychiatric history than the German/Swiss ones [t(51) = 2.3, p < 0.05]. Regarding gender distribution and the average duration of education, the German/Swiss and Romanian diagnostic groups were similar. The German/Swiss controls were significantly older than the Romanian ones [t(43) = 4.4, p < 0.001] and the German/Swiss patients with alcohol-related disorders were significantly younger than the Romanian ones [t(61) = 3.5, p < 0.001].

**Table 1:** Demographic characteristics of the control and of the patient groups^1, 2^

	Alcohol Related Disorders		Schizophrenic Disorders		Affective Disorders		Personality Disorders		Controls			
	
	G/S	R	G/S	R	G/S	R	G/S	R	G/S	R	Analysis	
	N		N		N		N		N		χ^2^	p
	
Gender											12	<.05
Female	6	10	10	11	14	19	11	6	23	15		
Male	14	15	18	13	10	11	15	9	15	10		
	Mean ± SD		Mean ± SD		Mean ± SD		Mean ± SD		Mean ± SD		F	p
	
Age	**31 ± 9**	**45 ± 12**	34 ± 8	36 ± 10	40 ± 12	44 ± 8	34 ± 8	32 ± 11	**38 ± 13**	**28 ± 7**	6	<.001
Education	2 ± 1	2 ± 1	3 ± 1	3 ± 1	3 ± 1	3 ± 1	2 ± 1	3 ± 1	3 ± 1	3 ± 1	4	<.01
Psychiatric history (yrs)	6 ± 1	7 ± 10	8 ± 9	11 ± 10	**4 ± 6**	**9 ± 9**	4 ± 5	6 ± 7	-	-	2	n.s.

^1 ^Abbreviations: G/S: German/Swiss; R: Romanian; Education: 0 = no education, 1 = school for the mentally challenged, 3 = middle school, 4 = high school;

^2 ^Areas where the values are written with bold characters indicate significant differences between German/Swiss and Romanian subjects within the diagnostic groups (i.e., alcohol-related disorders, schizophrenic disorders, affective disorders, personality disorders and control group respectively).

### Material

Life experiences were assessed with the Traumatic Antecedents Questionnaire (TAQ) [40]. The TAQ is a 42-item self-rating questionnaire, which covers 11 subscales enquiring into the severity of positive (i.e., competence and safety) and negative experiences (i.e., neglect, separation, secrets, emotional abuse, physical abuse, sexual abuse, witnessing, other traumas, and alcohol and drugs) during four developmental periods (ages 0–6, 7–12 13–18, and ≥ 19). Each subscale includes 2–6 items. Each item requires the occurrence of a certain type of experience for each of the different age periods. The subjects were asked to score on a frequency/intensity scale the degree to which it describes their experience: 0 ("never or not at all"), 1 ("rarely or a little bit"), 2 ("occasionally or moderately"), 3 ("often or very much"), and DK ("don't know"). In a subsequent step, the average scores were calculated within each developmental period for each of the 11 subscales. The procedure we used was the following: first, the "don't know" responses were noted in a non-numerical manner, by using asterisks (*) to indicate missing values and these values were counted as 0; secondly, the response scores were added up and the sum was divided by the total number of items within the subscale in that age period for which there were numerical scores. By using this procedure, we excluded "don't know" responses from the total scores calculation.

### Data analysis

Comparisons of demographic data were made with analysis of variance (ANOVA) and with two-tailed unpaired t-tests for continuous variables. Chi-square analysis was used to compare nominal data. The differences between groups were evaluated individually for each TAQ scale by repeated-measures ANOVA with the cultural background (German/Swiss versus Romanian), psychiatric status (alcohol-related disorders, schizophrenic disorders, affective disorders, personality disorders or controls), and gender (female versus male) as between-subjects factors, and developmental period (4 periods) as within-subjects factor. The probability level for rejecting the null hypothesis was set at P < 0.05. Post-hoc comparisons were carried out to evaluate main effects and interactions using Bonferroni/Dunn tests. A principal components analysis was also applied to the entire sample in order to identify those factors, which could account for individual variability across the eleven scales of the TAQ. The principal components were derived by using varimax rotation to orthogonalize solutions.

## Results

### Positive experiences

Table 2 lists group mean scores on each of the two positive experiences scales. The patients generally exhibited lower mean scores on reported positive experiences as compared to the controls. The reported level of *competence *did not differ between diagnostic groups [F(4,198) = 0.8, n.s] or cultural samples [F(1,198) = 0.1, n.s]. A main effect of developmental period [F(3,594) = 25.7, P < 0.001] was explained by the increase of competence from early childhood to adolescence (P < 0.05), and by the decrease of competence in adulthood as compared to adolescence (P < 0.05).

**Table 2:** Mean scores of positive experiences across developmental periods among all groups

Positive Experiences and Age at Onset	Alcohol Related Disorders	Schizophrenic Disorders	Affective Disorders	Personality Disorders	Control Group	Analysis	
	
	Mean ± SD	Mean ± SD	Mean ± SD	Mean ± SD	Mean ± SD	F	p
*Early Childhood (0–6)*							
Competence	1.5 ± 0.9	1.9 ± 0.9	1.7 ± 1.0	1.7 ± 0.9	1.7 ± 0.1	0.7	n.s
Safety	1.4 ± 0.8	1.7 ± 0.8	1.6 ± 0.8	1.2 ± 0.8	1.6 ± 0.7	3.2	<.05
*Latency (7–12)*							
Competence	2.1 ± 0.8	2.0 ± 0.8	2.1 ± 0.7	1.9 ± 0.8	2.1 ± 0.9	0.2	n.s
Safety	1.8 ± 0.8	1.8 ± 0.8	1.9 ± 0.7	1.3 ± 0.7	1.9 ± 0.7	5.1	<.001
*Adolescence (13–18)*							
Competence	2.1 ± 0.9	2.1 ± 0.7	2.2 ± 0.6	2.1 ± 0.8	2.3 ± 0.8	0.6	n.s
Safety	1.9 ± 0.8	1.7 ± 0.8	1.8 ± 0.8	1.3 ± 0.8	2.0 ± 0.8	4.0	<.01
*Adulthood (≥19)*							
Competence	1.9 ± 1.0	1.8 ± 0.8	2.1 ± 0.8	2.0 ± 0.8	2.2 ± 0.8	1.3	n.s
Safety	1.7 ± 0.8	1.7 ± 0.8	1.7 ± 0.8	1.5 ± 0.7	2.1 ± 0.7	4.9	<.001

For both cultural samples, patients with personality disorders reported lower values on the *safety *subscale than any of the other groups [F(4,215) = 4.5, P < 0.01]. Post hoc tests showed that patients with personality disorders (P < 0.001) and those with affective disorders (P < 0.01) reported less such experiences as compared to the controls. The reported level of safety increased through adolescence [F(3,645) = 11.1, P < 0.001], but the interaction of the developmental period with the psychiatric status revealed a decrease in safety accounts from the age of 13–18 years towards adulthood in all patient groups [F(12,645) = 2.8, P < 0.001].

### Negative experiences

Table 3 shows the mean scores of traumatic experiences for all patient groups and for the control group across developmental periods. Negative experiences were more frequent in patients than in controls as indicated by significant main effects of the psychiatric status on each of the nine subscales. In addition, there was an important increase of amount of reported negative experiences across developmental periods.

**Table 3:** Mean scores of negative experiences among all groups

Negative Experiences and Age at Onset	Alcohol Related Disorders	Schizophrenic Disorders	Affective Disorders	Personality Disorders	Control Group	Analysis	
	
	Mean ± SD	Mean ± SD	Mean ± SD	Mean ± SD	Mean ± SD	F	p
*Early Childhood (0–6)*							
Neglect	0.6 ± 0.5	0.7 ± 0.7	0.5 ± 0.5	0.8 ± 0.6	0.2 ± 0.3	8.1	<.001
Separation	0.5 ± 0.6	0.4 ± 0.5	0.4 ± 0.6	0.6 ± 0.8	0.2 ± 0.4	3.9	<.01
Secrets	0.9 ± 0.9	1.2 ± 1.1	1.0 ± 0.9	1.4 ± 1.1	0.6 ± 0.7	4.8	<.01
Emotional Abuse	0.7 ± 0.6	1.0 ± 1.0	0.7 ± 0.8	1.2 ± 0.9	0.3 ± 0.5	8.2	<.001
Physical Abuse	0.4 ± 0.6	0.4 ± 0.5	0.3 ± 0.5	0.7 ± 0.7	0.1 ± 0.3	5.7	<.001
Sexual Abuse	0.0 ± 0.3	0.2 ± 0.6	0.1 ± 0.3	0.3 ± 0.6	0.1 ± 0.4	2.0	n.s.
Trauma Witnessing	0.4 ± 0.5	0.4 ± 0.5	0.5 ± 0.6	0.7 ± 0.9	0.1 ± 0.2	7.6	<.001
Other Traumas	0.4 ± 0.5	0.3 ± 0.4	0.2 ± 0.4	0.4 ± 0.6	0.2 ± 0.3	3.1	<.05
Alcohol/Drug Abuse	0.4 ± 0.8	0.3 ± 0.6	0.5 ± 0.7	0.5 ± 0.7	0.1 ± 0.2	4.0	<.01
*Latency (7–12)*							
Neglect	0.7 ± 0.6	0.8 ± 0.7	0.7 ± 0.5	1.1 ± 0.8	0.5 ± 0.5	5.0	<.001
Separation	0.6 ± 0.7	0.5 ± 0.6	0.6 ± 0.7	0.9 ± 0.8	0.5 ± 0.6	2.8	<.05
Secrets	1.0 ± 0.9	1.2 ± 0.1	1.1 ± 0.9	1.6 ± 1.1	0.8 ± 0.8	4.2	<.01
Emotional Abuse	0.8 ± 0.6	1.1 ± 0.9	1.0 ± 0.8	1.4 ± 0.9	0.7 ± 0.8	4.6	<.01
Physical Abuse	0.5 ± 0.7	0.6 ± 0.7	0.6 ± 0.6	0.9 ± 0.8	0.5 ± 0.6	2.5	<.05
Sexual Abuse	0.1 ± 0.3	0.2 ± 0.5	0.1 ± 0.2	0.5 ± 0.8	0.1 ± 0.4	5.3	<.001
Trauma Witnessing	0.5 ± 0.6	0.5 ± 0.5	0.7 ± 0.6	0.9 ± 0.8	0.4 ± 0.5	5.5	<.001
Other Traumas	0.4 ± 0.5	0.4 ± 0.4	0.4 ± 0.5	0.5 ± 0.6	0.3 ± 0.4	1.4	n.s.
Alcohol/Drug Abuse	0.5 ± 0.7	0.3 ± 0.6	0.6 ± 0.7	0.6 ± 0.8	0.2 ± 0.4	4.1	<.01
*Adolescence (13–18)*							
Neglect	1.0 ± 0.6	0.9 ± 0.7	1.0 ± 0.5	1.2 ± 0.7	0.8 ± 0.6	2.5	<.05
Separation	0.9 ± 0.8	0.8 ± 0.8	0.9 ± 0.7	0.9 ± 0.7	0.6 ± 0.7	2.4	<.05
Secrets	1.1 ± 0.9	1.3 ± 1.0	1.1 ± 0.9	1.5 ± 1.0	0.7 ± 0.8	4.7	<.01
Emotional Abuse	0.8 ± 0.6	1.3 ± 0.9	1.1 ± 0.7	1.4 ± 0.9	0.8 ± 0.8	4.6	<.01
Physical Abuse	0.8 ± 0.7	0.5 ± 0.6	0.5 ± 0.6	1.0 ± 0.9	0.5 ± 0.6	5.0	<.001
Sexual Abuse	0.2 ± 0.4	0.2 ± 0.4	0.2 ± 0.4	0.5 ± 0.8	0.1 ± 0.3	3.2	<.05
Trauma Witnessing	0.6 ± 0.6	0.5 ± 0.5	0.7 ± 0.6	1.0 ± 0.8	0.4 ± 0.5	5.9	<.001
Other Traumas	0.6 ± 0.6	0.5 ± 0.5	0.4 ± 0.5	0.6 ± 0.5	0.3 ± 0.3	3.4	<.05
Alcohol/Drug Abuse	1.1 ± 1.0	0.6 ± 0.8	0.7 ± 0.9	0.9 ± 0.8	0.4 ± 0.7	5.3	<.001
*Adulthood *(19≥)							
Neglect	1.4 ± 0.8	1.3 ± 0.9	1.2 ± 0.7	1.2 ± 0.8	0.9 ± 0.7	3.0	<.01
Separation	1.6 ± 0.7	1.1 ± 0.9	1.5 ± 0.8	1.2 ± 0.9	1.0 ± 0.7	4.3	<.01
Secrets	1.1 ± 1.0	1.3 ± 1.1	1.2 ± 0.9	1.6 ± 0.9	0.5 ± 0.7	8.1	<.001
Emotional Abuse	0.9 ± 0.7	1.2 ± 0.9	1.1 ± 0.9	1.4 ± 0.9	0.6 ± 0.6	6.8	<.001
Physical Abuse	1.0 ± 0.9	1.0 ± 0.8	0.9 ± 0.9	1.0 ± 0.9	0.4 ± 0.6	5.1	<.001
Sexual Abuse	0.3 ± 0.6	0.6 ± 0.8	0.4 ± 0.7	0.5 ± 0.7	0.2 ± 0.4	2.8	<.05
Trauma Witnessing	0.8 ± 0.6	0.7 ± 0.7	1.0 ± 0.8	1.0 ± 0.9	0.5 ± 0.4	4.3	<.01
Other Traumas	1.2 ± 0.6	1.0 ± 0.7	1.1 ± 0.8	1.0 ± 0.6	0.4 ± 0.4	14.8	<.001
Alcohol/Drug Abuse	2.1 ± 0.8	0.8 ± 0.9	1.1 ± 0.9	1.0 ± 0.9	0.3 ± 0.6	29.3	<.001

**Figure 1:**
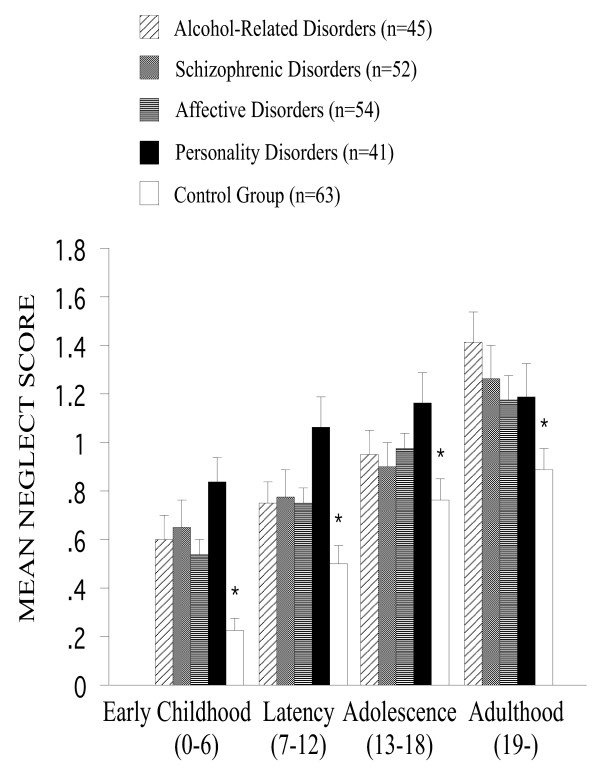
**Mean neglect score across developmental periods among all groups. **The psychiatric patients reported higher rates than the controls [F(4,214) = 5.7, P < 0.001]. There was an increase of the amount of reported neglect experiences across developmental periods [F(3,642) = 91.5, P < 0.001]. Error bars stand for standard error of the mean. Asterisks indicate significant main effects of the psychiatric status.

With respect to the experiences of *neglect*, the psychiatric patients reported higher rates than the controls [F(4,214) = 5.7, P < 0.001], the post hoc tests revealing that all patient groups reported more such experiences as compared to the controls: patients with personality disorders (P < 0.001), alcohol-related disorders (P < 0.05), schizophrenic disorders (P < 0.05), and affective disorders (P < 0.05). There was an increase of the amount of reported neglect experiences across developmental periods [F(3,642) = 91.5, P < 0.001]. Across developmental periods there were significant effects of the psychiatric status [F(12,642) = 3.2, P < 0.001]: the post hoc tests showed that patients with personality disorders (P < 0.001) and with alcohol-related disorders (P < 0.01) reported a highly significant increase of the amount of neglect experiences across developmental periods as compared to the controls (Figure 1).

Irrespective of the psychiatric status and developmental periods, Romanian subjects generally reported a higher amount of neglect experiences, as shown by the main effect of the cultural background [F(1,214) = 6.4, P < 0.05]. Romanian patients, particularly those with schizophrenic disorders, reported a higher incidence of neglect experiences than their German counterparts (P < 0.01), as revealed by the interaction between the psychiatric status and cultural background [F(4,214) = 5.6, P < 0.001]. As indicated by the interaction between the developmental period and the cultural background, the mean scores of neglect experiences were higher in the Romanian sample as compared to the German/Swiss one for the earliest (0–6 years) period [F(3,642) = 5.3, P < 0.001].

#### Separation

Patients, particularly those with alcohol-related disorders, personality disorders, and affective disorders reported more often separation experiences than controls [F(4,227) = 3.3, P < 0.01, P < 0.01 for post-hocs]. Mean scores on separation increased with age, and were highest in adulthood [F(3,681) = 103.0, P < 0.001].

#### Secrets

Higher patient mean scores were confirmed by the main effect of the psychiatric status [F(4,182) = 6.8, P < 0.001], especially for those with personality disorders (P < 0.001) and with schizophrenic disorders (P < 0.001), as revealed by post-hoc tests. There was also an indication of sensitivity to the cultural background [F(1,182) = 7.5, P < 0.01], as there was an increase in these scores in the Romanian sample, irrespective of the diagnosis and developmental period.

**Figure 2:**
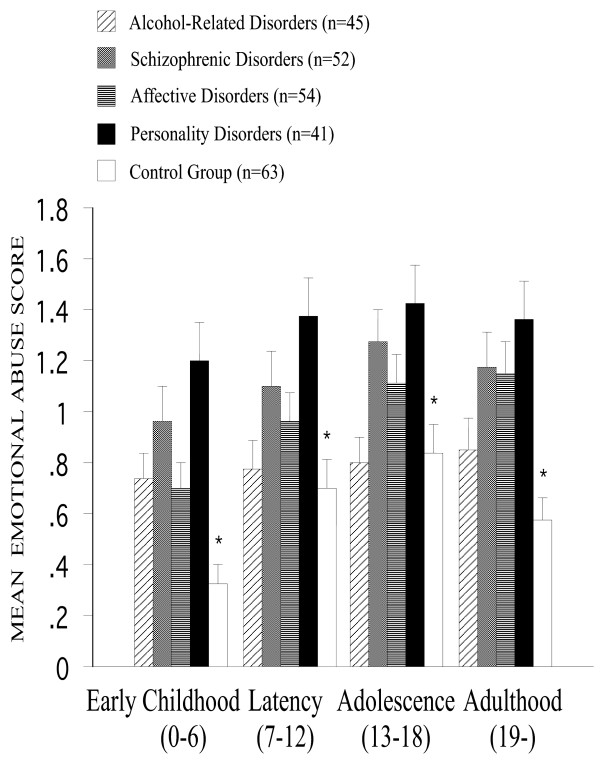
**Mean emotional abuse score across developmental periods among all groups. **Emotional abuse was more frequently reported by patients than by controls [F(4,194) = 7.0, p < 0.001]. A main effect of the developmental period [F(3,582) = 24.0, P < 0.001] was explained by an increase of the reported emotional abuse from early childhood to adolescence, and a decrease in adulthood were noted. Error bars stand for standard error of the mean. Asterisks indicate significant main effects of the psychiatric status.

*Emotional abuse *was more frequently reported by patients than by controls [F(4,194) = 7.0, P < 0.001] and more frequently by patients with personality disorders (P < 0.01) and by schizophrenic patients (P < 0.05) than the ones with a history of alcohol-related disorders (Figure 2). A main effect of the developmental period [F(3,582) = 24.0, P < 0.001] was explained by an increase of the reported emotional abuse from early childhood to adolescence (P <0.05), and a decrease in adulthood (P < 0.05) were noted. Similar to the case of the neglect experiences, the Romanian sample scored also higher than the German/Swiss sample, mainly for the earliest (0–6 yr.) period, as revealed by the interaction between the development period and the cultural background [F(3,582) = 5.4, P < 0.01].

**Figure 3:**
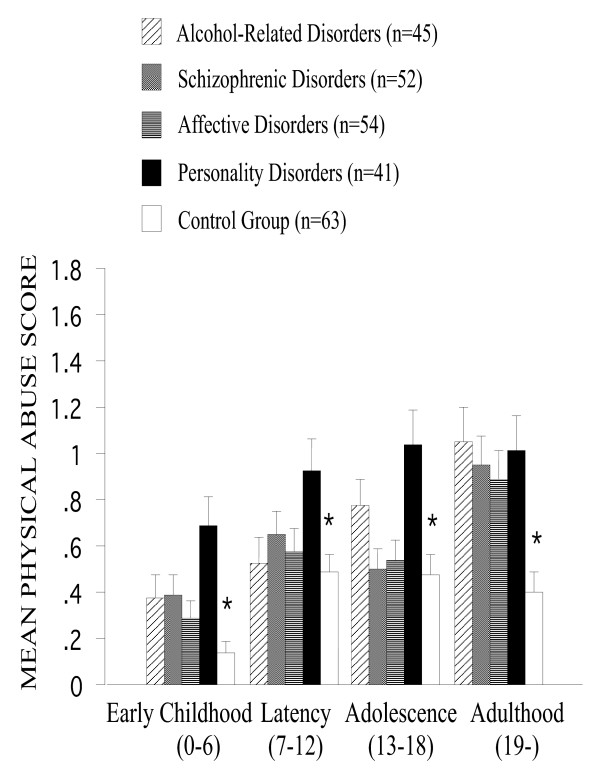
**Mean physical abuse score across developmental periods among all groups. **Physical abuse was more often reported by patients with personality disorders [F(4,202) = 5.7, P < 0.001]. The reports of physical abuse generally increased across developmental periods, with adulthood as the most susceptible period of such reports [F(3, 606) = 35.1, P < 0.001]. Error bars stand for standard error of the mean. Asterisks indicate significant main effects of the psychiatric status.

Irrespective of the developmental period, *physical abuse *was more often reported by patients with personality disorders [F(4,202) = 5.7, P < 0.001] (Figure 3). Post-hoc comparisons also revealed higher rates of physical abuse reports among patients with alcohol-related disorders (P < 0.01) and with schizophrenic disorders (P < 0.05) than among controls. The reports of physical abuse generally increased across developmental periods, with adulthood as the most susceptible period of such reports [F(3, 606) = 35.1, P < 0.001]. The interaction of the developmental period with the psychiatric status showed that this increase in physical abuse reports across developmental periods was mainly to be remarked in patients [F(12,606) = 3.0, P < 0.001].

**Figure 4:**
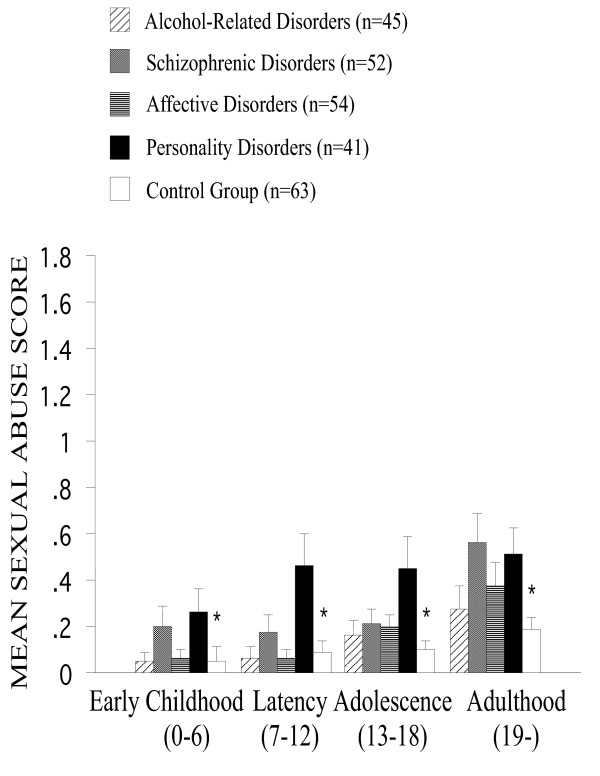
**Mean sexual abuse score across developmental periods among all groups. **Sexual abuse was primarily reported by patients, and not by controls, and particularly by patients with personality disorders [F(4,205) = 5.2, P < 0.001]. If sexual abuse was experienced, it occurred particularly in later developmental periods [F(3,615) = 20.4, P < 0.001]. Error bars stand for standard error of the mean. Asterisks indicate significant main effects of the psychiatric status.

*Sexual abuse *(Figure 4) was primarily reported by patients, and not by controls [F(4,205) = 5.2, P < 0.001], and particularly by patients with personality disorders (P < 0.001). Higher rates of sexual abuse were reported among patients with alcohol-related disorders (P < 0.01), with schizophrenic disorders (P < 0.05), and with affective disorders (P < 0.05) than among controls as shown by post-hoc tests. If sexual abuse was experienced, it occurred particularly in later developmental periods [F(3,615) = 20.4, P < 0.001]. Sexual abuse was more often experienced by female patients [F(1,205) = 10.0, P < 0.001] after puberty [F(3, 615) = 10.0, P < 0.001], as revealed by the interaction between the developmental period and gender. We also found a 3-way interaction between the developmental period, psychiatric status and cultural background [F(12,615) = 2.6, P < 0.01). The interaction between the developmental period and cultural background revealed that Romanian but not German/Swiss schizophrenics reported more frequently sexual abuse particularly in adulthood [F(3,615) = 5.0, P < 0.01].

*Trauma witnessing *was reported most often by patients with personality disorders as compared to all other groups [F(4,209) = 8.0, P < 0.001]. Post-hoc tests showed that patients with affective disorders (P < 0.01) and with alcohol-related disorders (P < 0.05) also reported more experiences of trauma witnessing than the controls. Irrespective of the diagnosis, Romanian patients, but not controls, reported higher mean scores on this variable and more often than their German/Swiss counterparts [F(1,209) = 17.0, P < 0.001]. The interaction between the cultural background and developmental period indicated in the Romanian sample an increase of trauma witnessing in adulthood [F(3,627) = 8.0, P < 0.001].

#### Other traumas

Similar to the pattern of trauma witnessing, all patients reported a greater number of traumatic events than the control group [F(4,211) = 8.0, P < 0.001]: alcohol-related disorders (P < 0.001), personality disorders (P < 0.001), schizophrenic disorders (P < 0.01), and affective disorders (P < 0.01), as explained by post-hocs. An increase in the amount of other traumas reports across the developmental periods with highest values in adulthood for all patient groups [F(12, 633) = 7.0, P < 0.001] was also revealed.

#### Alcohol and drug abuse

As previously expected, patients treated for alcohol-related disorders reported more alcohol and drug abuse than all the other groups [F(4,213) = 12.3, P < 0.001]. The post-hoc tests showed that the other patient groups also reported more alcohol and drug abuse when compared to the control group: affective disorders (P < 0.001), personality disorders, (P < 0.001) and schizophrenic disorders (P < 0.05). As also anticipated, abuse increased across developmental periods until adulthood [F(3,639) = 110.0, P < 0.001], particularly in patients with alcohol-related disorders, as revealed by the interaction between the developmental period and psychiatric status [F(12,639) = 14.3, P < 0.001]. Irrespective of the diagnosis, Romanian patients, but not controls, showed higher mean scores on reporting alcohol and drug abuse than their German/Swiss counterparts [F(4,213) = 3.4, P < 0.01], as shown by the interaction between the psychiatric status and cultural background. Compared to the German group, alcohol and drug abuse in the Romanian sample was higher, particularly in adulthood [F(3,639) = 9.8, P < 0.001].

### Interrelations

A principal components factor analysis was performed to explore interrelationships among TAQ subscales. The results of this analysis indicated that the most appropriate solution involved five factors that jointly accounted for 56.2% of the total variance in the dataset. Table 4 summarizes the results of the varimax rotation for the five-factor solution. The first factor showed high positive loadings on *physical abuse*, *sexual abuse*, *trauma witnessing*, *and other traumas*, obviously explains the traumatic experiences. The second factor showed high positive loadings on *competence *and *safety*, apparently accounting for variance attributed to positive experiences. The third factor showed high positive loadings on the *first year of illness *with *alcohol and drug abuse*. The fourth factor, consisting of *separation*, evidently explains disruptions of attachment. The fifth factor, which included *secrets *and *emotional abuse*, appeared to account for family chaos. Thus, the structure of the study instrument was well reproduced for the present sample, which included different psychiatric diagnoses and different cultural backgrounds.

**Table 4:** Varimax solution with five factors for negative and positive childhood experiences across developmental periods in psychiatric patients with different diagnoses^1^

	Factor Loading^2^				
	
Variables	FACTOR 1: Traumatic Experiences^3^	FACTOR 2: Positive Experiences^4^	FACTOR 3: Vulnerability to Alcohol Abuse^5^	FACTOR 4: Disruptions of Attachment^6^	FACTOR 5: Family Chaos^7^
Competence	-0.0	**0.8**	0.2	0.1	0.1
Safety	0.2	**0.8**	-0.0	0.0	-0.2
Neglect	0.2	-0.3	0.1	-0.0	0.3
Separation	0.4	0.2	-0.0	**0.8**	0.1
Secrets	-0.0	-0.1	-0.0	0.1	**0.7**
Emotional Abuse	0.2	0.0	0.6	-0.0	**0.6**
Physical Abuse	**0.7**	-0.0	-0.0	0.0	0.0
Sexual Abuse	**0.4**	0.0	0.0	-0.6	0.1
Witnessing	**0.6**	0.0	-0.0	0.0	0.2
Other Traumas	**0.6**	0.2	0.1	0.2	0.1
Alcohol & Drug Abuse	0.4	-0.2	**0.5**	0.0	-0.3
First Year of Illness	-0.1	0.1	**0.8**	-0.1	0.2

^1 ^Total percent of variance = 56.2%

^2 ^Shaded areas indicate specific domains of the TAQ contributing to each factor

^3 ^Eigenvalue = 4.88; percent of variance = 40.7%

^4 ^Eigenvalue = 1.476; percent of variance = 12.3%

^5 ^Eigenvalue = 1.013; percent of variance = 8.4%

^6 ^Eigenvalue = 0.835; percent of variance = 7.0%

^7 ^Eigenvalue = 0.815; percent of variance = 6.8%

## Discussion

The study aimed at exploring whether psychiatric diagnoses, e.g. alcohol-related disorders, schizophrenic disorders, affective disorders, and personality disorders are related to retrospectively reported positive and negative life events across developmental periods, and if so, whether special developmental periods are characterized by more negative experiences than others.

Our findings demonstrate a strong association between reports of traumatic events and certain psychiatric disorders. In other studies, negative experiences were reported by individuals with diagnoses such as affective disorders [18,41] and schizophrenic disorders [42,43], but these experiences were less common and cumulatively less severe. Negative experiences were particularly prominent in patients with personality disorders [24,25,44] and in patients with substance-related disorders [26,45,46]. Negative experiences were reported more often in late childhood and adolescence than in early childhood and adulthood. Previous studies indicated that the earlier onset of abuse was associated with greater severity and longer duration of mental problems [2,10,45,47].

If the present findings are consistent with some prior studies [5,9,16] in that they indicate a relationship between physical and sexual abuse and psychiatric disorders, they do not support the view expressed by Van der Kolk et al. about early abuse at an early stage of development [48]. The current investigation showed that many psychiatric patients had terrible histories of childhood physical and/or sexual abuse. This finding was marginally significant for the childhood sexual abuse histories and must therefore be interpreted with caution. However, one should keep in mind that self-report questionnaires depend heavily upon conscious retrieval capacity for autobiographic events. It is conceivable that in the current group of patients, early abuse events were less remembered as compared to abuse events experienced later in childhood. An advantage of the TAQ used in the present study is the assessment of negative experiences during both childhood and adulthood, while most of the other studies have so far focused primarily on the impact of childhood abuse, except Cascardi et al. [49] and Goodman et al. [32]. Another advantage of the TAQ is that it addresses the issue of neglect [50]. Given the sample of patients with different psychiatric diagnoses, this replicates Van der Kolk's et al. notion that patients who experience neglect early in their lives develop serious problems with affect regulation [51]. The present data add to the evidence, suggesting that neglect, emotional and physical abuse are experienced by many psychiatric patients [52,53]. This implies that although childhood traumas may contribute to a mental disorder in adulthood, the lack of secure attachments maintains it. Although emotional neglect has received less attention, perceived emotional rejection by parents has been associated with alcohol abuse [54] and delinquency [55] during adolescence and adulthood. Early emotional injuries could possibly trigger vulnerability to noxious experiences.

Furthermore, experiences of parental loss or separation were prominent in adulthood especially for the patients with alcohol-related disorders and with affective disorders. The high incidence of such negative experiences during this period in the patients with alcohol-related disorders could be, at the same time, a direct consequence of the behavioral deviance of these individuals and contribute to the maintenance of alcohol abuse.

### Limitations of the study

The present data has to be considered in the light of several possible limitations. First, the information obtained by self-report and without external evidence could be less reliable and valid, especially if we take into account the sensitive nature of this research. Herman and Schatzow, however, provide empirical support for the validity of abused patients' self-reports as well [56]. They found that when corroborating evidence is sought, the majority of women are able to obtain confirmation of abuse. No independent corroborating evidence was sought for any self-reported case of childhood negative experiences. Therefore, the validity of abuse reports cannot be assured. Recall may be biased, but there is no evidence that psychiatric patients are more likely to lie about or imagine child abuse [57,58]. There is some evidence, however, that "patients are biased to underreport abuse histories rather than to over report them" [59]. There were some "don't know" subject answers regarding abuse/neglect experiences, most of them in the early childhood. Most probably, the patients had difficulties recalling experiences that occurred at a very young age rather than trying to evade giving a positive answer. Furthermore, another methodological limitation in this study is that measuring neglect/emotional abuse in early childhood is particularly difficult as the awareness of it necessitates the development of a degree of differentiation and autonomy, which is seldom the case with psychiatric patients.

Both individual interviews and self-report questionnaire methods present higher figures than chart reviews do, indicating that patients usually do not spontaneously offer such information to their therapists. When offered, the information is not reliably documented [57]. However, the data from our ongoing study in patients with personality disorders suggest that reports on events in general and physical abuse events in particular are highly stable across two measurement periods of time separated by 24 months. We also note that our sample consisted of psychiatric inpatients, and thus may not be representative of the broader population of patients with these disorders. The clinical validity of the TAQ has also been criticized [60]. The questionnaire is meant to be an applied clinically oriented measure, which has not yet been proved to be a psychometrically sound research instrument. This issue should be addressed in future studies using both convergent and divergent instruments.

Romanian patients diagnosed with schizophrenic disorders differed significantly, with respect to the number of negative events, as compared to their German counterparts. One factor accounting for this difference might be the stressful environment during the former Ceausescu regime in Romania. During this 25-year period violations of human rights, terror, and corruption prevailed [61,62]. This result may also be due to the different diagnostic procedures used by Romanian and German/Swiss clinicians. Reports of higher rates of psychotic-like or specifically schizophrenic symptoms do not necessarily imply a diagnosis of schizophrenia. Once abuse is identified, a change of diagnosis, from schizophrenia to PTSD, is often made, with significant advantages for the individuals [30].

## Conclusions

The present study demonstrates an association between negative life events in childhood and psychiatric diagnoses in adult life, which is in line with a number of other studies [6,63]. Unlike previous reports [3,64], we found that psychiatric patients were more likely to report more negative life events during late childhood and adolescence rather than during early childhood and adulthood. These conclusions corroborate with one of the central hypothesis of life-span psychotraumatology, that is, adolescence is an extremely critical phase in the development of later psychopathology [65,66]. However, in line with findings offered by earlier controlled studies [37,38], psychiatric patients were more likely to report higher rates of negative life events during childhood than controls did.

Although one cannot assume a direct causal relationship between childhood abuse and adult psychopathology from the present data, the present study provides further preliminary and explorative evidence for the high load of negative life events in psychiatric patients. An advantage of this study is the examination of the abuse histories across a range of four psychiatric diagnoses within a controlled comparison design. Our findings are important and clinically highly relevant for further etiological research of causal and maintenance factors of psychiatric symptomatic, as well as for the research on the treatment of these conditions.

The special value of the study lies in its cross-national comparison from a clinical psychological point of view including a highly underresearched country like Romania. More attention should be paid to the sad situation of the patients in Romania who are often under inadequate pharmacological and insufficient psychotherapeutic treatment, as well as under inappropriate hospitalization conditions. Further research should concentrate on the epidemiology and developmental psychopathology of psychiatric populations in other countries than the usually researched ones. Generally, reports of traumatic experiences during the whole lifespan should be more carefully considered in the clinical diagnosis process and in the development of treatment programs for the psychiatric patients.

## Competing interests

The author(s) declare that they have no competing interests.

## Authors' contributions

ES carried out the study in Germany, performed the statistical analysis and drafted the manuscript. DB carried out the study in Romania and drafted the manuscript. BR conceived of the study and drafted the manuscript. FN participated in the design of the study. MS participated in the design of the study. KS participated in the coordination of the study. KH participated in the coordination of the study. TE conceived of the study and drafted the manuscript. All authors read and approved the final manuscript.

## Pre-publication history

The pre-publication history for this paper can be accessed here:


